# Structural Insights into the Mechanical Behavior of Large-Area 2D Covalent Organic Framework Nanofilms

**DOI:** 10.1021/acsami.5c03512

**Published:** 2025-04-18

**Authors:** Luana Gazzato, Elena Missale, Daniele Asnicar, Francesco Sedona, Giorgio Speranza, Alessandra Del Giudice, Luciano Galantini, Alberta Ferrarini, Marco Frasconi, Maria F. Pantano

**Affiliations:** † Department of Chemical Sciences, University of Padova, via Marzolo 1, 35131 Padova, Italy; ‡ Department of Civil, Environmental and Mechanical Engineering, University of Trento, via Mesiano 77, 38123 Trento, Italy; § Fondazione Bruno Kessler, via Sommarive 18, 38123 Trento, Italy; ∥ Istituto di Fotonica e Nanotecnologie & Consiglio Nazionale delle Ricerche IFN—CNR, via alla Cascata 56/C Povo, 38123 Trento, Italy; ⊥ Department of Industrial Engineering, University of Trento, via Sommarive 9, 38123 Trento, Italy; # Department of Chemistry, Sapienza University of Rome, P.le A. Moro5, 00185 Rome, Italy

**Keywords:** 2D COF, nanomechanics, freestanding nanofilm, membranes, interfacial polymerization

## Abstract

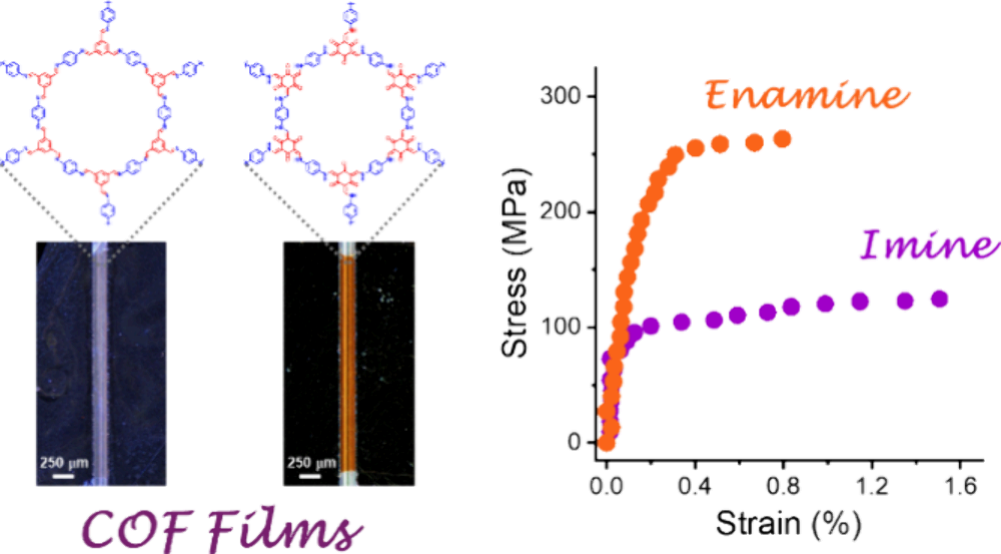

Two-dimensional covalent organic frameworks (2D COFs) are periodic, permanently porous, lightweight solids with remarkable structural modularity, enabling precise control over their properties. As thin films, they have shown promising applications in chemical separations and organic electronics, making it crucial to understand their stability under mechanical stress. Here, we investigate how two different chemical linkages commonly used for 2D COFs, specifically imine and enamine, influence the mechanical properties of nanoscale thick films. Centimeter-scale 2D COF films with a thickness below 100 nm were synthesized by a condensation reaction at a liquid–liquid interface and subsequently transferred onto patterned substrates for mechanical testing. By employing a custom-made nanotensile testing platform, we achieved a comprehensive mechanical characterization of freestanding 2D COF films over a large area (0.5 mm^2^), a size relevant for device applications. The enamine-linked COF exhibits a higher Young’s modulus and tensile strength but a lower fracture strain compared to the imine-linked COF, a difference attributed to the tightly stacked structure of the enamine-linked COF, as confirmed by molecular dynamics simulations. This distinct mechanical behavior reveals a fundamental relationship between the linkage chemistry of 2D COF and their mechanical properties, providing valuable insights that can drive the development of strong and durable thin-film devices based on 2D COFs.

## Introduction

Two-dimensional (2D) covalent organic frameworks (COFs), characterized by covalently bonded organic monomers extending in two orthogonal directions,^1,2^ have emerged as lightweight and strong porous polymeric materials for a wide variety of applications,^3^ including catalysis,^4,5^ gas separation,^6^ energy storage,^7,8^ and sensors.^9^ Aromatic rigid monomers linked via dynamic covalent reactions, such as imine condensation^10^ or boronate ester metathesis,^11^ are commonly used to generate 2D COFs, with the geometry of the monomer unit dictating the pore shape and size of the resulting structure. The two-dimensional sheets of 2D COFs are held together through noncovalent forces, including van der Waals, aromatic stacking, and hydrogen bonding interactions.^12−14^ In addition, changing the side chain functional groups to the COF building blocks enables the modulation of the interlayer interactions. For example, incorporating methoxy side chain units has been shown to enhance the chemical stability of imine-based COFs by forming strong interlayer hydrogen bonds.^4,15^ The formation of intramolecular hydrogen bonds between imine nitrogen and hydroxyl groups in β-ketoenamine-linked COFs has been reported to enhance molecular rigidity, leading to improved emission properties of the COFs.^16^ Therefore, controlling interlayer interactions in 2D COFs presents an effective strategy for tuning key material properties,^12,14^ such as thermal and charge/electrical conductivity, gas adsorption and separation, as well as mechanical strength and stretchability, ultimately achieving specific performance targets or desired applications.

However, a comprehensive understanding of the mechanical properties of 2D COFs and their relationship to different linkage chemistries, topologies, and monomer structures is still far from being achieved despite their importance in ensuring the reliability and stability of these materials, particularly when utilized as nanometer thick films in various applications.^17^ To date, most studies relied on the computational investigation of monolayer 2D COFs.^18,19^ Using density functional theory (DFT) and molecular dynamics (MD) simulations, a theoretical strength of up to 27.9 GPa was predicted for a perfect monolayer COF.^19^ In addition, large-scale MD simulations provided crucial insights into how structural defects affect the mechanical properties of COFs.^20,21^ Recently, DFT calculations investigating interlayer interactions in few-layer 2D COFs have revealed a significant increase in sliding energy between adjacent layers due to the formation of hydrogen bonds facilitated by methoxy side chains.^22^ This led to an enhancement in the Young’s modulus of the 2D COF films, as demonstrated also through mechanical characterization of the synthesized films.

In experimental studies, the Young’s modulus of 2D COF nanofilms is typically derived by employing techniques such as atomic force microscopy (AFM) point load measurements,^22^ buckling instability,^23^ or, to a more limited extent, tensile tests.^24^ While the former techniques revealed effectiveness in the evaluation of the Young’s modulus of ultrathin films (<100 nm), they rely on indirect estimation and do not enable a comprehensive mechanical characterization, including failure processes, that are strongly related to the interlayer sliding of the 2D sheets. On the contrary, tensile testing can provide more comprehensive information, including the fracture properties of the tested material samples.^24,25^ However, obtaining defect-free, freestanding specimens on a large area remains challenging for films with nanometer thickness.^26^

**Figure 1:**
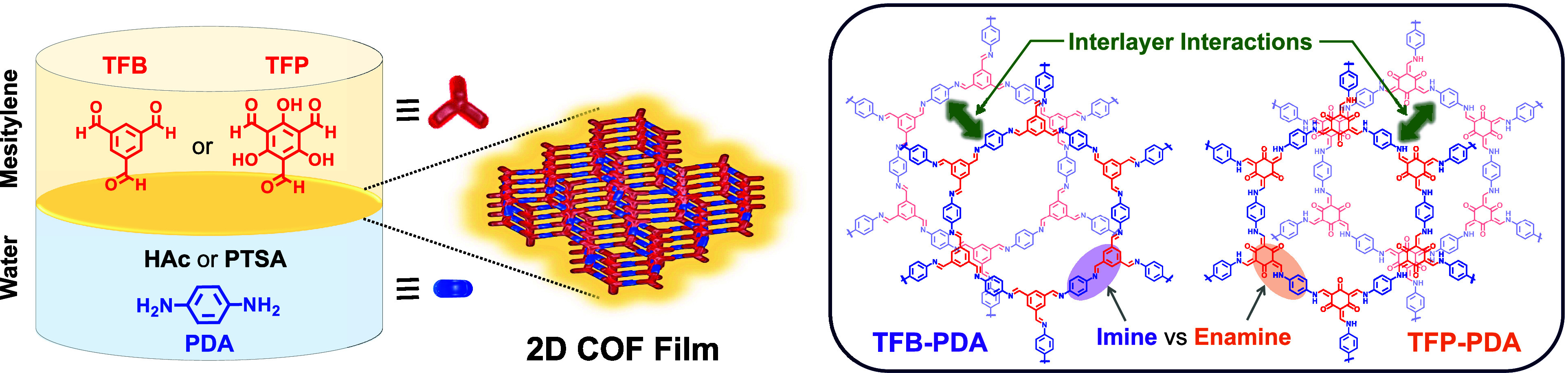
Schematic representation of the condensation reaction at a mesitylene–water interface of diamine monomer, PDA, with trialdehyde monomer, TFB or TFP, to provide an imine-linked TFB-PDA COF and a β-ketoenamine-linked TFP-PDA COF, respectively.

Here, we report two large-area, freestanding 2D COF nanofilms featuring imine and ketoenamine linkers, with the aim of gaining insights into the relationship between the structure of 2D COFs and their mechanical properties. In particular, the synthesized COFs consist of *p*-phenylenediamine (PDA) linked to benzene-1,3,5-tricarbaldehyde (TFB) or 2,4,6-triformylphloroglucinol (TFP) to yield TFB-PDA COF and TFP-PDA COF, respectively (Figure 1). Both COFs exhibit an identical hexagonal porous topology due to the geometry of the monomers. However, they present significant chemical differences arising from the distinct bonds formed between the PDA monomer and the two different trialdehyde monomers, which also influence the stacking of the 2D layers. The straightforward synthesis routes we pursued, based on Schiff condensation reaction at a mesitylene–water interface, resulted in the fabrication of centimeter-scale films with controlled thicknesses below 100 nm. These nanofilms were characterized as freestanding through a custom-made tensile testing platform. This allowed us to derive a complete set of mechanical properties for both COFs across a large area. Our findings reveal the high stiffness and strength of COF nanofilms, highlighting their potential for applications requiring high mechanical performances.

## Results and Discussion

**Figure 2:**
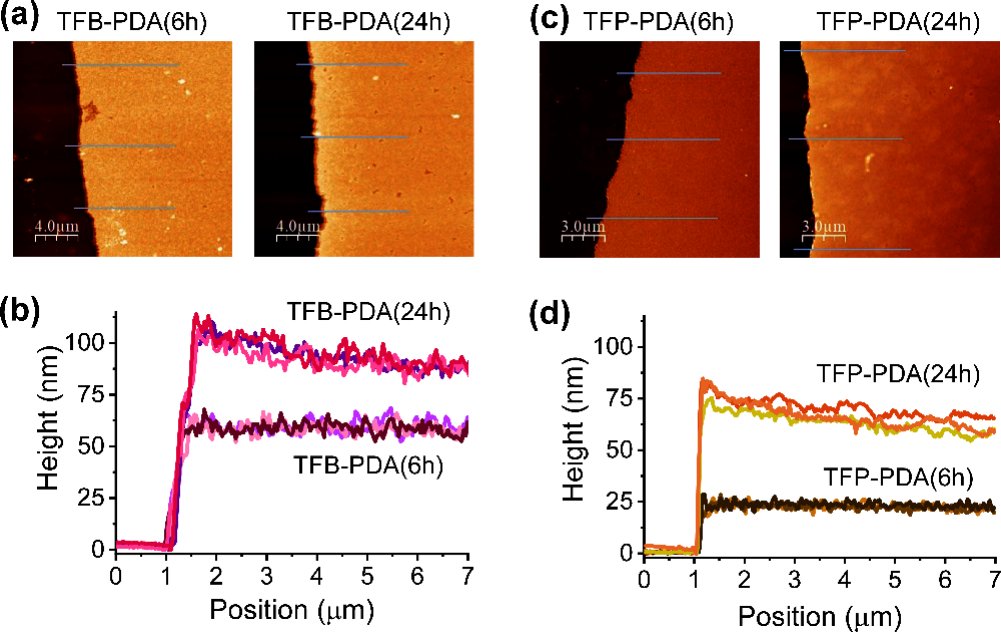
Tapping-mode AFM height images of the TFB-PDA (a) and TFP-PDA (c) COF films on silicon substrates after 6 and 24 h of reaction. Thickness profiles of the TFB-PDA (b) and TFP-PDA (d) films along the blue lines.

Figure 1 illustrates the synthesis of the targeted 2D COF nanofilms at the liquid–liquid interface, using methylene and water as solvents, which are compatible with the solubilities of the aldehyde and amine monomers, respectively. Specifically, two 2D COFs were synthesized, featuring the same diamine monomer, 4-phenylenediamine (PDA), and two aldehyde monomers, triformylbenzene (TFB) or 1,3,5-triformylphloroglucinol (TFP), to provide an imine-linked TFB-PDA COF and a β-ketoenamine-linked TFP-PDA COF, respectively. The aldehyde monomer, TFB or TFP, was solubilized in the mesitylene phase, while the amine monomer, PDA, was solubilized in the aqueous phase. To optimize the syntheses of the COF films, the reaction progress was followed by using a digital camera, and the resulting films were characterized by atomic force microscopy (AFM) after being transferred from the reactor to a silicon substrate. Due to the low solubility of TFB and TFP in water, their reaction with PDA occurred exclusively at the methylene-water interface, where contact with the acid catalyst promoted the formation of a smooth film. Acetic acid (HAc) or *p*-toluene sulfonic acid (PTSA) were used as Bro̷nsted acid catalysts. Both acids were initially tested in both the organic and aqueous phases, leading to significant differences in film growth and ultimately impacting film quality. Specifically, we found that PTSA more effectively controls the formation of the TFP-PDA film, while acetic acid proves to be more effective in the synthesis of TFB-PDA COF. This finding is consistent with previous studies on the interfacial synthesis of imine and β-ketoenamine-linked COF films.^17,27^ For the synthesis of TFB-PDA COF, film formation was observed within 24 h when HAc was used in either the organic or aqueous phase (Figure S1), whereas no extended film formed within the same time frame when PTSA was used. AFM characterization of the TFB-PDA films transferred onto silicon substrates revealed significant differences in thickness and film quality depending on whether HAc was used in the organic or aqueous phase. When HAc was added to the organic phase, a continuous film with a thickness of 75 nm formed; however, a significant number of particulates remained on the surface, even after extensive washing of the film (Figure S2). In contrast, when HAc was added in the aqueous phase at a concentration of 9 mM alongside PDA (1.5 mM), with TFB (2 mM) in the organic phase, a uniform and continuous film formed at the interface. After 6 h of reaction, the TFB-PDA film reached a thickness of approximately 56 nm, further increasing to 87 nm after 24 h (Figure 2a,b). AFM analysis also revealed that, under these conditions, complete film formation was achieved with nanoscale roughness of 2.8 nm after 6 h and 4.7 nm after 24 h, indicating that these conditions are optimal for the synthesis of smooth and continuous TFB-PDA COF films.

For the synthesis of TFP-PDA, adding HAc to the organic phase led to the rapid formation of large orange TFP-PDA COF particulates, which settled at the bottom of the vessel within just 1 h of reaction (Figure S3). When HAc was added into the aqueous phase, the organic phase turned orange more slowly, with COF particulates appearing after approximately 3 h (Figure S4). AFM analysis of the transferred films revealed significant defects in both cases, including many micrometer-sized particulates on the surface (Figure S5). By contrast, the reaction with PTSA proceeded much more slowly and did not generate a significant number of particulates, even after 24 h (Figure S4). The optimized reaction conditions, PTSA (9 mM) in the aqueous phase, combined with PDA (0.6 mM) and TFP (0.8 mM) in the organic phase, yielded a smooth, defect-free TFP-PDA film, as confirmed by AFM (Figure 2c). The film transferred on the silicon substrate after 6 h of reaction, had a thickness of 23 nm, which increased to approximately 70 nm after 24 h (Figure 2d), with surface roughness values of 1.6 and 5.2 nm, respectively. For all subsequent experiments, we utilized the TFB-PDA and TFP-PDA COF films synthesized after 24 h of reaction, as this condition produced uniform and continuous films covering the entire reactor surface (Figure S6).

**Figure 3:**
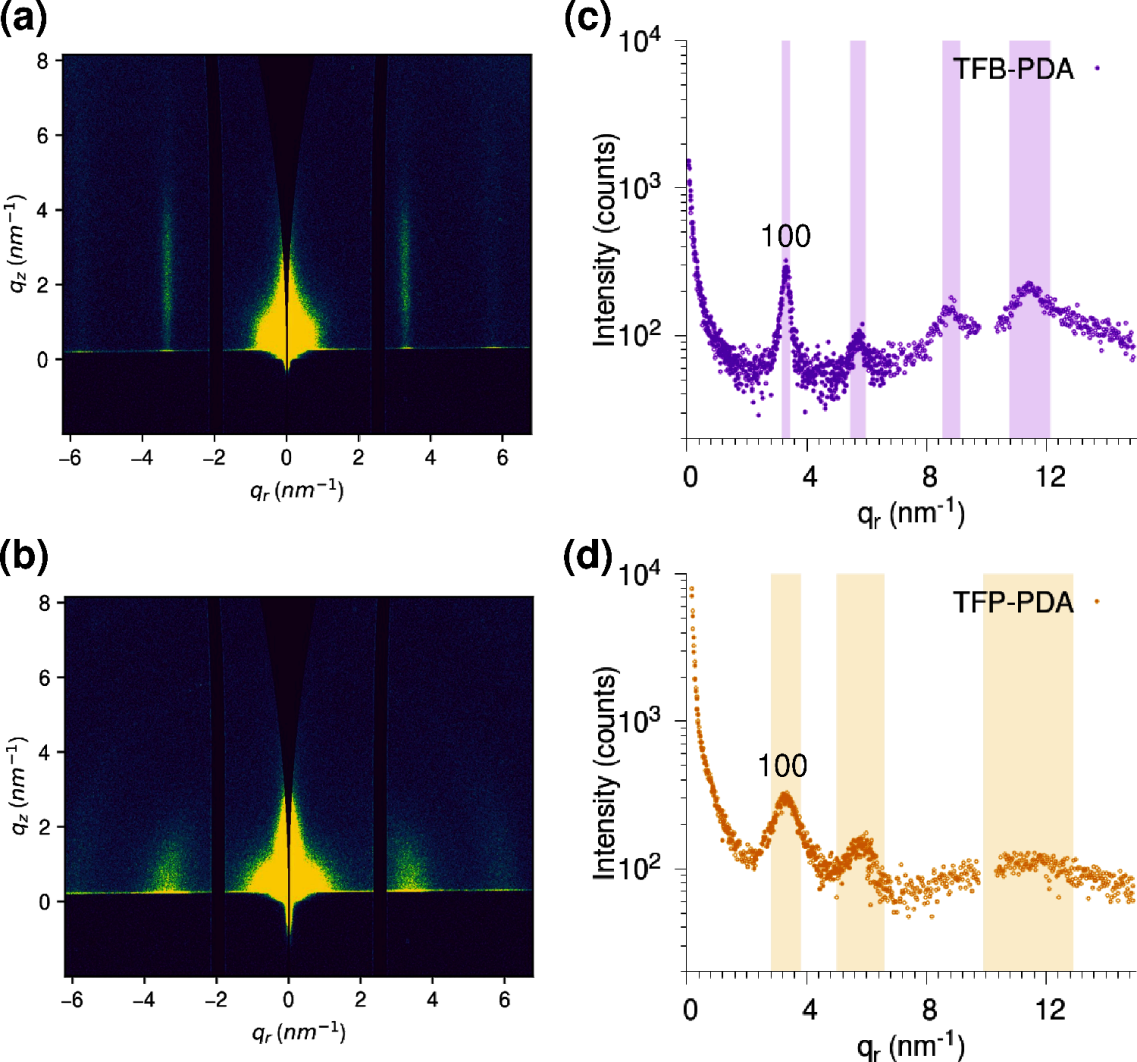
2D GISAXS images (a, b) and in-plane intensity profiles (c, d) of the TFB-PDA (a, c) and TFP-PDA (b, d) films.

Scanning electron microscopy (SEM) images further confirm the formation of continuous TFB-PDA and TFP-PDA COF films with uniform thickness over large areas (Figure S7). In particular, SEM images reveal smooth surfaces for both COF films after transfer onto silicon substrates, with no evidence of cracks, indicating the success and reliability of preparation and transfer procedures. The crystalline nature of the TFB-PDA and TFP-PDA films was confirmed by 2D grazing incidence small-angle X-ray scattering (GISAXS), as shown in Figure 3. The characteristic in-plane distance between adjacent pores, determined from the position of the first diffraction peak given by the (100) planes, was 1.9 nm for both COF films, consistent with previously reported structures^6,28^ (see also Table S1). In both cases, the GISAXS patterns indicate that the COF films were preferentially oriented with their *c*-axis perpendicular to the substrate. The TFB-PDA film exhibited a significantly higher degree of in-plane order, featuring a highly oriented 2D pattern with sharper peaks, indicative of large, coherently oriented crystalline domains (Figure 3c). In addition, even if the limited GISAXS angular range challenged the detection of crystalline order in the surface-normal direction (where stacking distances below 1 nm are expected), a broad maximum only was observed in out-of-plane cuts at *q* > 10 nm^–1^ (Figure S8). This broad band suggests limited structural coherence across the film thickness as probed by grazing incidence, indicating a degree of offset stacking between the COF nanosheets.^29^

**Figure 4:**
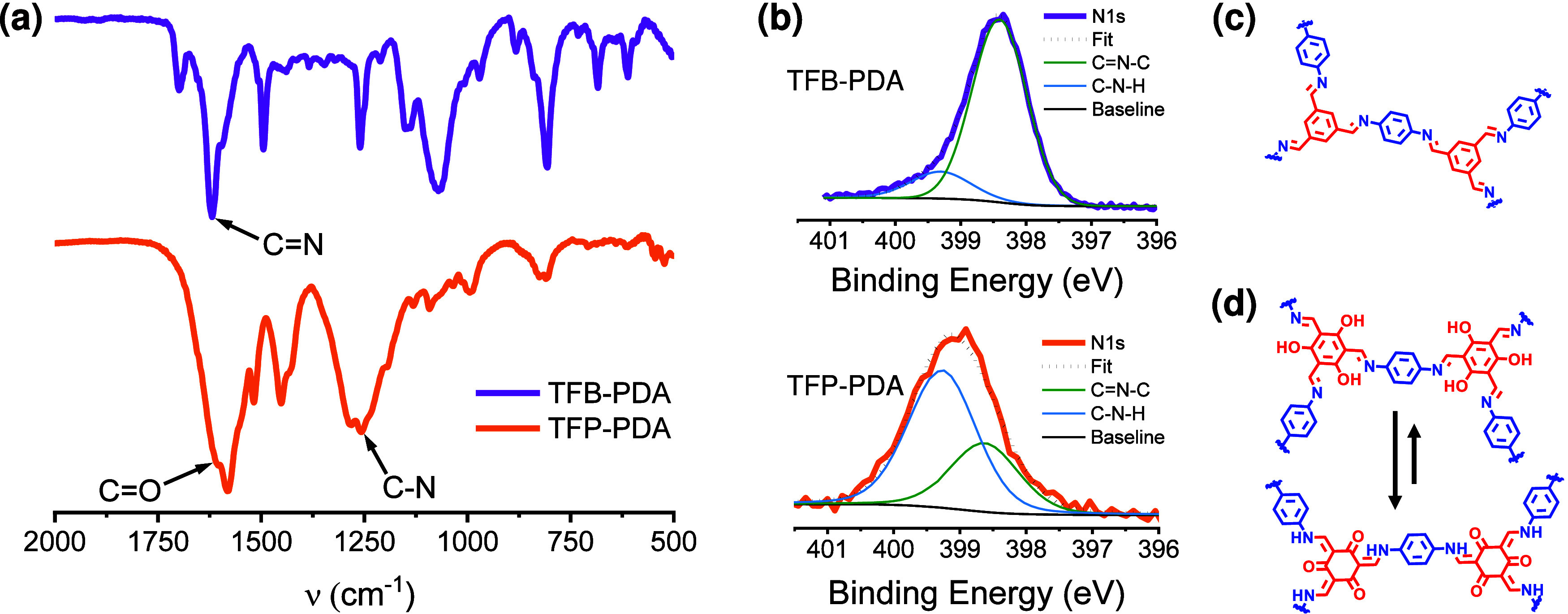
Spectroscopic characterization of the COF nanofilms. FT-IR spectra (a), XPS N 1s spectra (b) and structures of TFB-PDA (c) and TFP-PDA (d).

The chemical composition of the COF films was analyzed using Fourier-transform infrared spectroscopy (FTIR) and X-ray photoemission spectroscopy (XPS). The formation of the imine bond (C=N) in TFB-PDA was confirmed by the presence of a peak at 1618 cm^–1^, while the N–H and C=O signals from the PDA and TFB monomers, observed at 3100–3400 and 1696 cm^–1^ respectively, were absent in the FTIR spectra of the COF (Figures 4a and S9a). Besides, UV/vis spectra of the TFB-PDA COF film deposited on quartz slides showed two absorbance bands at 390 and 298 nm, characteristic of imine compounds (Figure S10a). The XPS spectra further confirm imine formation, as evidenced by the N 1s peak fitting (Figures 4b and S11), which reveals a primary component at 398.5 eV corresponding to imine nitrogen (C=N–C). A minor peak at 399.5 eV is observed, likely corresponding to a small amount of unreacted amino groups on the COF surface.

In the FTIR spectrum of the TFP-PDA COF (Figures 4a and S9b), the complete disappearance of the N–H stretching bands from the PDA monomer is observed, along with the appearance of a peak at 1257 cm^–1^ corresponding to the C–N bond, confirming the occurrence of the Schiff base reaction. Additionally, the shift of the C=O peak from 1649 cm^–1^ in the TFP spectrum to 1603 cm^–1^ in the COF spectrum indicates the tautomerism associated with this reaction. The UV–vis spectrum of the TFP-PDA COF film revealed a prominent absorption band at 480 nm with a shoulder at 520 nm (Figure S10b), which was significantly red-shifted compared to the absorption of imine compounds. XPS analysis (Figure 4b) provided deeper insights into the chemical bonding of this COFs, confirming the formation of a keto-enamine equilibrium (Figure 4d). The N 1s spectrum of the TFP-PDA COF (Figure 4b) exhibited a peak at approximately 398.5 eV, similar to TFB-TDA, which corresponds to imine nitrogen (C=N–C) and is associated with the enol-imine tautomer. Additionally, a more intense peak around 399.5 eV was observed, indicative of enamine formation (C–N–H). The higher intensity of the last peak indicates that the reaction favored the keto form, with a smaller fraction of the enol form attributable to the imine product. This interpretation is further supported by the analysis of the O 1s spectrum for TFP-PDA (Figure S12), which revealed two components at 532.1 and 530.2 eV, corresponding to hydroxyl oxygen (C–OH) and ketone oxygen (C=O), respectively. These findings confirm the presence of a keto–enol equilibrium within the TFP-PDA COF. Previous studies on β-ketoenamine-linked COFs using XPS^30^ and solid-state Nuclear Magnetic Resonance (NMR) spectroscopy^31^ have identified the coexistence of both tautomeric forms—the keto-enamine and enol-imine—though their precise relationship is not always well-defined. In our study, the integration of the O 1s and N 1s XPS peaks indicates that the keto-enamine form is predominant, with an approximate 2:1 ratio over the enol-imine form.

The permanent porosity of the synthesized COFs was confirmed by N_2_ gas adsorption–desorption measurements at 77 K (Figure S13). The calculated Brunauer–Emmett–Teller (BET) surface areas (*S*_BET_) were 371 m^2^/g for TFB-PDA and 308 m^2^/g for TFP-PDA. Nonlocal density functional theoretical isotherm analysis revealed a defined pore size distribution centered at 1.77 and 1.62 nm for TFB-PDA and TFP-PDA COFs. These values are consistent with those reported for other COFs with similar structures,^28,32^ and confirm that the porous architecture of our 2D COFs is stable and remains accessible to penetrants.

**Figure 5:**
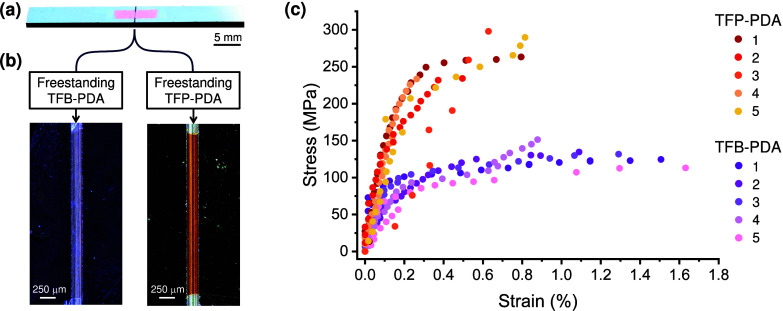
Mechanical characterization of freestanding 2D COF films. (a) Photographic image of a COF nanofilm transferred onto silicon prenotched slices used for the mechanical characterization. (b) Optical microscopy images showing the TFB-PDA and TFP-PDA COF films freestanding over the topside prenotch (∼200 μm wide) on the silicon slice. Elsewhere, the film adheres to the silicon substrate. (c) Stress–strain curves of TFB-PDA and TFP-PDA COF film specimens (five samples tested for each COF, obtained from five independent reaction batches).

The mechanical characterization of the COF films was conducted using a custom-made nanotensile testing platform (Figure S14).^33^ The experiment involved transferring the COF film floating on water onto a silicon slice with a prenotch on the topside with a width of approximately 200 μm (Figure 5a). This setup ensured the film remained freestanding in the notched region while adhering to the silicon substrate elsewhere. Optical microscopy images of the TFB-PDA and TFP-PDA COF films on silicon slices confirmed the presence of intact, crack-free freestanding films, demonstrating successful specimen preparation (Figure 5b). This configuration resulted in a testing area of the nanofilm of ∼0.5 mm^2^. Figure 5c presents the stress–strain curves for TFB-PDA and TFP-PDA films. The TFB-PDA film exhibited a tensile strength of 131 ± 14 MPa, a strain at failure of 1.3 ± 0.3%, and a Young’s modulus of 61 ± 14 GPa. In contrast, the TFP-PDA film demonstrated a significantly higher tensile strength of 264 ± 30 MPa, a lower strain at failure of 0.6 ± 0.2%, and a higher Young’s modulus of 99 ± 29 GPa. These results indicate that TFP-PDA is twice as strong and 1.5 times stiffer than TFB-PDA, while TFB-PDA can accommodate nearly twice the strain before failure, yet both COFs exhibit a similar area under their stress–strain curves, with values of 1.1 ± 0.6 MJ/m^3^ for TFP-PDA and 1.3 ± 0.3 MJ/m^3^ for TFB-PDA.

The Young’s modulus of our TFP-PDA and TFB-PDA films falls within the range of values reported in the literature for other imine-linked COF nanofilms. For example, a Young’s modulus of 89.1 ± 3.8 GPa was recently derived from AFM deflection tests carried out on a composite film of Sc_2_O_3_ and 2D COF with a thickness of ∼11 nm, synthesized at a liquid–liquid interface via the condensation reaction of 1,4-phthalaldehyde (TPA) and amino 5,10,15,20-tetrakis(4-aminophenyl)-porphyrin (TAPP) monomers.^34^ A crystalline monolayer of polyimine was reported to have a Young’s modulus of 267 ± 30 GPa, as determined by buckling metrology,^23^ while AFM deflection tests conducted on a 4.7 nm thick TTA-DHTA COF film (TTA: 4,4′,4″-(1,3,5-triazine-2,4,6-triyl)trianiline; DHTA: 2,5-dihydroxyterethaldehyde) revealed a Young’s modulus of 25.9 ± 0.6 GPa.^35^ AFM deflection tests conducted on three-layer COF films, synthesized via Schiff-base condensation of 1,3,5-tris(aminophenyl)benzene (TAPB) and 1,4-phenylenedialdehyde (PDA) or 2,5-dimethoxyterephthaldehyde (DMTP), reported 2D elastic moduli of 41.6 ± 1.7 and 50.4 ± 2.2 N/m, respectively.^22^ Additionally, an imine-linked COF film (∼50 nm thick) composed of TAPB and DHTA was characterized using a MEMS-based tensile testing device, revealing a Young’s modulus of 10.38 ± 3.42 GPa.^24^ Recently, tensile testing of polycrystalline 2D polyimine films (∼19 nm thick) composed of TAPP and 2,5-dihydroxyterephthalaldehyde monomers revealed a fracture strain of 6.5% and Young’s modulus of 8.6 ± 2.5 GPa.^36^ These tensile tests on COFs reported a higher fracture strain compared to our findings. While these differences in mechanical properties can certainly be attributed to differences in COF structure, synthesis, and transfer methods, another key factor is the significantly smaller size of the films tested in these works (in the order of hundreds of μm^2^ or lower) compared to ours (∼0.5 mm^2^). Indeed, films with a larger area can be more prone to structural defects, contributing to reducing the fracture strain. However, testing films over large-areas provides a more realistic assessment of their mechanical behavior under practical conditions, particularly in applications such as membranes and thin-film devices. Notably, in a recent study, we used the same tensile testing setup employed here to characterize TAPB-PDA COF films with a thickness of 85 nm, which exhibited comparable tensile strength (188 ± 57 MPa) and strain at failure (1.0 ± 0.3%) to the TFB-PDA films in this study, but with a lower Young’s modulus (37 ± 15 GPa).^37^ For comparison, it is important to note that while TAPB-PDA COF films feature imine linkages like the TFB-PDA films in this study, they have a larger pore size than TFB-PDA.^37^

**Figure 6:**
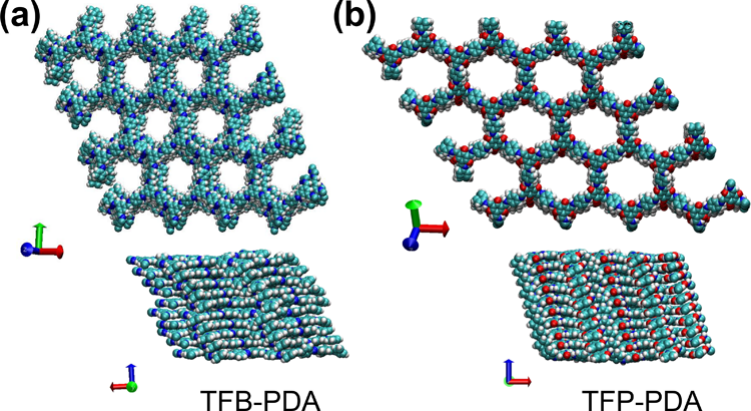
Snapshots of MD simulations of 10-layer stacks of TFB-PDA (a) and TFP-PDA (b), shown in longitudinal (top) and lateral (bottom) views.

To gain molecular-level insights into the differences in the mechanical behavior of the TFB-PDA and TFP-PDA COF films observed during testing, we performed Molecular Dynamics (MD) simulations. MD simulations were carried out with the all-atom OPLS-AA force field^38^ (see Experimental Section for details). Figure 6 presents longitudinal and lateral views of the structures obtained from MD simulations of 10-layer stacks for both COFs. Although both COFs present similar topologies, the enamine-linked COF (TFP-PDA) is more compact than the imine-linked COF (TFB-PDA). This observation is further supported by the analysis of lateral displacements between adjacent layers (Figure S16), which reveals that TFP-PDA COF exhibits smaller lateral displacements with narrower fluctuations compared to TFB-PDA COF, which suggests a more tightly stacked structure.

The different arrangement characterizing the two COFs can be primarily attributed to differences in interlayer electrostatic interactions, which are significantly stronger in TFP-PDA, particularly due to the C=O (+0.36*e* on C, −0.48*e* on O) and N–H (−0.24*e* on N, +0.27*e* on H) groups. Intralayer hydrogen bonding is prevalent in TFP-PDA, with carbonyl oxygens and adjacent nitrogens serving as hydrogen bond acceptors and donors, respectively. In contrast, interlayer hydrogen bonding is significantly less probable for both COFs, likely due to geometric constraints that hinder O···H–N formation. However, in TFP-PDA, O–H···N=C hydrogen bonds may still occur in the presence of its enol tautomer.

In summary, this work demonstrates that the mechanical properties of large-area 2D COF thin films are strongly influenced by their chemical linkages, specifically imine and enamine bonds. While previous studies have explored the mechanical properties of COFs, they have primarily focused on individual COF structures^23,24,36^ or comparisons among COFs with the same linkage and topology but differing monomer functionalities.^22^ Moreover, most of these studies have been related to small-scale films (with areas on the order of hundreds of μm^2^) or relied on indirect measurements, making it difficult to obtain a thorough understanding of the mechanical properties relevant to thin-film device applications. Here, by employing a custom-made nanotensile testing platform and a robust transfer method, we achieved a full mechanical characterization of two different freestanding 2D COF films with nanoscale thickness over device-relevant length scales (0.5 mm^2^ testing area). Our tensile tests reveal distinct mechanical properties of the two COF films, with a significant difference between the stiff, strong enamine-linked COF (TFP-PDA) and the more deformable imine-linked COF (TFB-PDA). These findings not only deepen our understanding of the structure–mechanical property relationship in COFs but also open new opportunities for large-area 2D COF films in structural and functional applications, particularly in flexible optoelectronic devices,^39^ coatings and membranes,^40^ where both scalability and mechanical robustness are essential.

## Conclusions

In conclusion, this work provides key insights into the structure–mechanical property relationship of 2D COF films with nanoscale thickness, which is crucial in developing high-performance yet robust devices. Centimeter-scale COF films were fabricated via interfacial synthesis, yielding high-quality films with uniform nanometer-level thickness and crystallinity. Large-area COF films were successfully transferred as freestanding films onto a custom-designed silicon substrate, overcoming a major challenge in mechanical testing and also providing a valuable approach for integrating 2D COFs into devices. Tensile testing reveals a distinctive role of the chemical linkage on the mechanical properties of the two COF films. Specifically, TFP-PDA COF exhibited a tensile strength of 264 ± 30 MPa and Young’s modulus of 99 ± 29 GPa, while TFB-PDA demonstrated a higher strain at failure, reaching 1.3 ± 0.3%. The tightly stacked structure of the enamine-linked COF (TFP-PDA) in comparison to imine-linked COF (TFB-PDA), as evidenced by MD simulations, likely explains the limited deformation capability observed in TFP-PDA relative to TFB-PDA. Unlike other 2D materials, such as graphene, where stacking interactions are difficult to control,^12^ COFs provide a chemically tunable platform in which the selection of monomers and linkages allows precise control over porosity, as well as optical and electronic properties. This work demonstrates that the mechanical properties of 2D COF can be effectively controlled through structural design, laying the foundation for rational optimization of the mechanical performance of 2D COFs and enabling their integration into next-generation thin-film technologies.

## Experimental Section

### Materials

All reagents were purchased from commercial suppliers and used without further purification. Benzene-1,3,5-tricarbaldehyde (TFB, 98%) and 2,4,6-triformylphloroglucinol (TFP, 98%) were purchased from TCI Europe N.V. *p*-phenylendiamine (PDA, 98%), acetic acid (HAc) and *p*-toluenesulfonic acid monohydrate (PTSA), mesytilene and methanol were all purchased from Sigma-Aldrich Co.

### Synthesis of COF Films

#### TFB-PDA COF Film

29.2 mg of PDA was dissolved in 180 mL of milli-Q water, followed by the addition of 94 μL of pure HAc (final concentrations: PDA 1.5 mM, HAc 9 mM). 25 mL of this solution was poured into a Teflon custom-made trough (diameter Ø = 6 cm). Subsequently, 3 mL of a 2 mM solution of TFB in mesitylene was carefully layered onto the aqueous phase using a microliter syringe. After the reaction at room temperature for 24 h, a thin yellow film was observed at the liquid–liquid interface.

#### TFP-PDA COF Film

11.7 mg of PDA was dissolved in 180 mL of milli-Q water, followed by the addition of 308.2 mg of PTSA (final concentrations: PDA 0.6, PTSA 9 mM). 25 mL of this stock solution was then added in a Teflon custom-made trough (diameter Ø = 6 cm). Subsequently, 3 mL of a 0.8 mM solution of TFP in mesitylene was slowly layered onto the aqueous phase using a microliter syringe. The reaction proceeded for 24 h at room temperature, yielding an orange film at the liquid–liquid interface.

### Transfer of COF Films

After the polymerization of the COF film, the reactor was placed in a larger crystallizing dish, and milli-Q water was slowly added using a pipet until the film reached the top of the reactor. Concurrently, the larger container was also filled with water to a level that exceeded the reactor’s height. This facilitated the floating of the COF film on the water surface of the larger dish. The COF film floating on water was then collected with a glass slide, gently rinsed with methanol and water, and transferred to a large crystallizing dish filled with water. Finally, the film was collected with a suitable substrate, quartz, silicon, or silicon prenotched slices for the mechanical characterization.

### Mechanical Characterization

Double prenotched silicon slices (3 cm in length and 5 mm in width) with a topside notch of ∼200 μm width were used to place the COF film for the tensile test. These were conducted with a custom-made tensile testing platform (Figure S14).^33^ This platform includes a piezo motor-driven linear stage (AG-LS25 by Newport) with a minimal incremental displacement of 50 nm, a custom-made spring with load sensing function (load sensor) engraved from 1 mm thick poly(vinyl chloride) foil, and a double prenotched silicon slice positioned between them. The silicon slice supports the specimen to be tested and is broken into two facing blocks using a custom-made clamp before testing. In this setup, a small amount of pressure is sufficient to create a tiny fracture line running from the bottom prenotch to the top prenotch of the silicon slice. Once this fracture line propagates, the actuator and spring are attached to each side of the now-separated silicon slice blocks, and the clamp is released. The platform is then positioned under an optical microscope. During the tensile test, the actuator applies displacement steps of approximately 350 nm to the specimen; part of the delivered displacement is required to deform the specimen, and another part is transferred to the load sensor spring that deforms, too. As a consequence of this process, the fracture line opens and is recorded at each step. At the end of the test, when the specimen fails, all collected images are processed with a custom routine in the Matlab environment to calculate the displacement of each side of the broken silicon slice, with one corresponding to the specimen end connected to either the load sensor spring or the actuator. The load sensor and the specimen act like a series of springs and experience the same load, calculated by multiplying the load sensor displacement (i.e., the displacement of the silicon block connected to it) by its calibrated spring constant. The stress is calculated as the ratio of the force measured by the load sensor to the cross-sectional area of the specimen. Specimen strain is determined as the ratio between the relative displacement of the silicon blocks and the width of the top prenotch of the silicon slice (∼200 μm), which corresponds to the specimen gauge length.

### Grazing-Incidence Small-Angle X-ray Scattering

The grazing-incidence small-angle X-ray scattering (GISAXS) was performed using a Xeuss 2.0 Q-Xoom system (Xenocs SAS, Grenoble, France), equipped with a microfocus Genix 3D X-ray Cu source (λ = 0.1542 nm, Cu Kalpha radiation) and a two-dimensional Pilatus3 R 300 K detector (Dectris Ltd., Baden, Switzerland). The incidence angle α_i_ between the beam and the sample surface used is 0.175°, below the critical angle of the silicon support, as verified by preliminary X-ray reflectivity scans (Figure S8). The beam size is collimated to have a cross-section of 0.0625 mm (vertical, out of sample plane) × 1 mm (horizontal, in sample plane). Scattering patterns are collected with sample–detector distances of 330 and 262 mm and a total acquisition time of 8 h per sample.

### Sorption Measurements

Nitrogen adsorption and desorption isotherms were performed at 77 K with an ASAP 2460 Surface Area and Porosity Analyzer (Micromeritics Instrument Corporation). Surface areas were calculated using the Brunauer–Emmett–Teller (BET) adsorption model applied between *P*/*P*_0_ values of 0.04 and 0.16. Pore size distributions were determined using the nonlocalized density functional theory (NLDFT) model. All samples were degassed for 12 h at 90 °C under vacuum before the gas adsorption analyses.

### UV/Vis Absorption Spectroscopy

UV/vis Absorption spectra were recorded using a Cary 60 Agilent spectrophotometer. To record the optical absorption spectra, COF films were transferred onto quartz substrates.

### FT-IR Spectroscopy

Fourier transform infrared spectra (FT-IR) were obtained with a FT-IR Nicolet Nexus 670 spectrometer. All samples were prepared as KBr pellets. Twenty-five scans were collected with a resolution of 2 cm^–1^.

### X-ray Photoemission Spectroscopy

Measurements were performed at room temperature using a VG Scienta XM 650 X-ray source. The X-rays produced were monochromatized using a VG Scienta XM 780 monochromator optimized for Al Kα radiation (1486.7 eV). Photoelectrons were collected and analyzed with a Scienta SES 100 electron analyzer. The peak position was calibrated by shifting the principal component of the C 1s to 284.8 eV, and all other peaks in the spectrum were calibrated accordingly.

### Atomic Force Microscopy

Atomic force microscopy (AFM) images were taken using an Agilent 5500 Atomic Force Microscope system operating in tapping mode.

### Scanning Electron Microscopy

Scanning electron microscopy (SEM) images were taken on a Zeiss Sigma HD microscope equipped with a Schottky FEG source, one detector for backscattered electrons, and two detectors for secondary electrons (InLens and Everhart Thornley).

### Molecular Dynamics Simulations

Molecular dynamics (MD) simulations were performed using the all-atom OPLS-AA force field,^38^ which has been demonstrated to be reliable through comparisons with results obtained at the density functional theory (DFT) level.^21,41−43^ OPLS parameters were obtained for fragments using the online generator LibParGen,^44^ and some parameters were adjusted to comply with the periodicity of the systems. The atomic coordinates of the unit cells, available in the CURATED database^45,46^ (11020N2 for TFB-PDA and 12000N2 for TFP-PDA), were used to generate the initial structures (Figure S15) and assign partial atomic charges. The simulations were performed using the GROMACS package (version 2022).^47^ The equations of motion were integrated using the leapfrog algorithm with a time step of 0.5 fs. Stacks of 10 2 × 2 supercells were constructed and placed in a box with periodic boundary conditions, with a size sufficiently large to prevent interactions with the images of the 2D COF along the stacking direction. Long-range electrostatic interactions were treated using the smooth particle-mesh Ewald (PME) method.^48^ Cutoffs for short-range electrostatic and van der Waals interactions were both set to 1 nm. After energy minimization, a 100 ps trajectory in the *NVT* ensemble was run, followed by a 1.1 ns simulation in the *NPT* ensemble. A final 100 ps *NVT* trajectory was used for sampling and analysis. The temperature was maintained at 300 K using the V-rescale thermostat,^49^ and the Parrinello–Rahman barostat^50^ was used with the pressure set to 1 bar. Trajectory analysis was performed using GROMACS tools and homemade codes. Molecular visualization was carried out with the VMD^51^ and iRASPA^52^ packages.

## Supporting Information Available

Characterization of the COF film’s growth, GISAXS, FT-IR, UV–vis, and XPS characterizations of COF films, nitrogen adsorption analysis, mechanical characterization, molecular dynamics simulations (PDF)
