# Supplementary material for: Structural Insights into the Mechanical Behavior of Large-Area 2D Covalent Organic Framework Nanofilms
Source: ACS Appl Mater Interfaces. 2025 Apr 18;17(17):25819–27. doi: 10.1021/acsami.5c03512 (PMC12051174; doi:10.1021/acsami.5c03512)
Supplement: Supplementary file 1 — am5c03512_si_001.pdf [file am5c03512_si_001.pdf]

Supporting Information

Structural Insights into the Mechanical Behavior of Large-Area 2D Covalent Organic Framework Nanofilms

Luana Gazzato,[§] *Elena Missale,*[†,○] *Daniele Asnicar,*[§] *Francesco Sedona,*[§] *Giorgio Speranza,*[‖,Δ,⊥] *Alessandra Del Giudice,*[‡] *Luciano Galantini,*[‡] *Alberta Ferrarini,*[§] *Marco Frasconi,**[,§] *Maria F. Pantano*[*,†]

[§]Department of Chemical Sciences, University of Padova, via Marzolo 1, 35131 Padova, Italy.

[†]Department of Civil, Environmental and Mechanical Engineering, University of Trento, via Mesiano 77, 38123 Trento, Italy.

[‖] Fondazione Bruno Kessler, via Sommarive 18, 38123 Trento, Italy.

[Δ]Istituto di Fotonica e Nanotecnologie & Consiglio Nazionale delle Ricerche IFN—CNR, via alla Cascata 56/C Povo, 38123 Trento, Italy.

[⊥]Department of Industrial Engineering, University of Trento, via Sommarive 9, 38123 Trento, Italy.

[‡]Department of Chemistry, Sapienza University of Rome, P.le A. Moro5, 00185 Rome, Italy.

*Emails: marco.frasconi@unipd.it; maria.pantano@unitn.it

Table of Contents

A. Growth of COF Nanofilms……………………………………………………………S2
B. GISAXS……………...……………...……………...…………………………………...S6
C. FT-IR Spectroscopy……………..………..………………………………..………...S8
D. UV-Vis Spectroscopy………………………………………………………………..….S8
E. X-ray Photoemission Spectroscopy…………………………………………..........S9
F. Nitrogen Adsorption Analysis………………………..………………………..……...S10
G. Mechanical Characterization……………………...…………………...………...S11
H. Molecular Dynamics Simulations ……………………...………………...….S11
I. References..……………………..……………………………...…………………..….S12

A. Growth of COF Nanofilms

The progress of the reaction was monitored using a digital camera. To optimize the reaction conditions, we employed transparent glass reactors, which allowed for clear observation of the reaction progress. Once the reaction conditions were established, we carried out the synthesis in a Teflon reactor; the chemically inert properties of Teflon ensured that large-scale, uniform COF films grew at the water/mesitylene interface without adhering to the container walls. The prepared films were characterized using AFM after being transferred from the reactor to a silicon substrate.

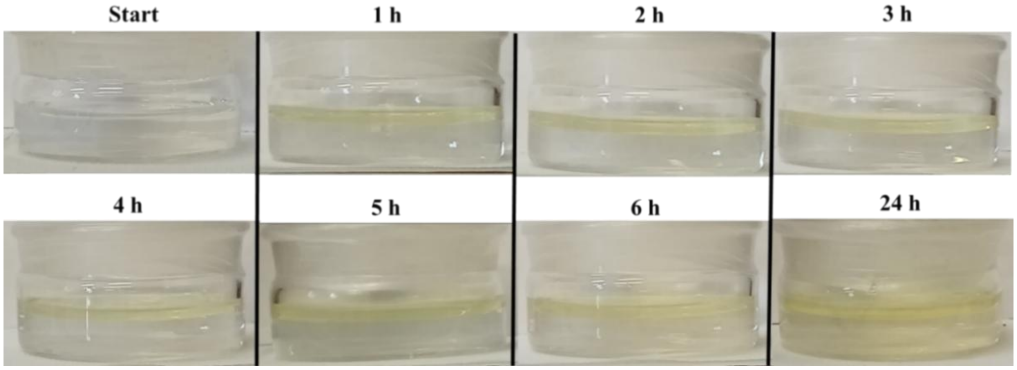

Figure S1. Photographic images showing the growth of TFB-PDA COF films. HAc was introduced in the aqueous phase along with the diamine monomer, PDA, while the TFB was present in the organic phase.

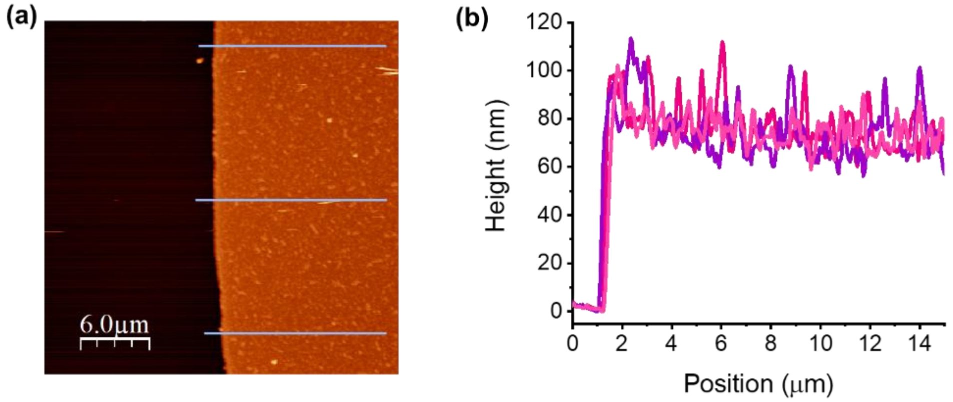

Figure S2 Tapping-mode AFM height image (a) and thickness profiles (b) of the TFB-PDA film deposited on a silicon substrate after 24 h of reaction using HAc in the organic phase.

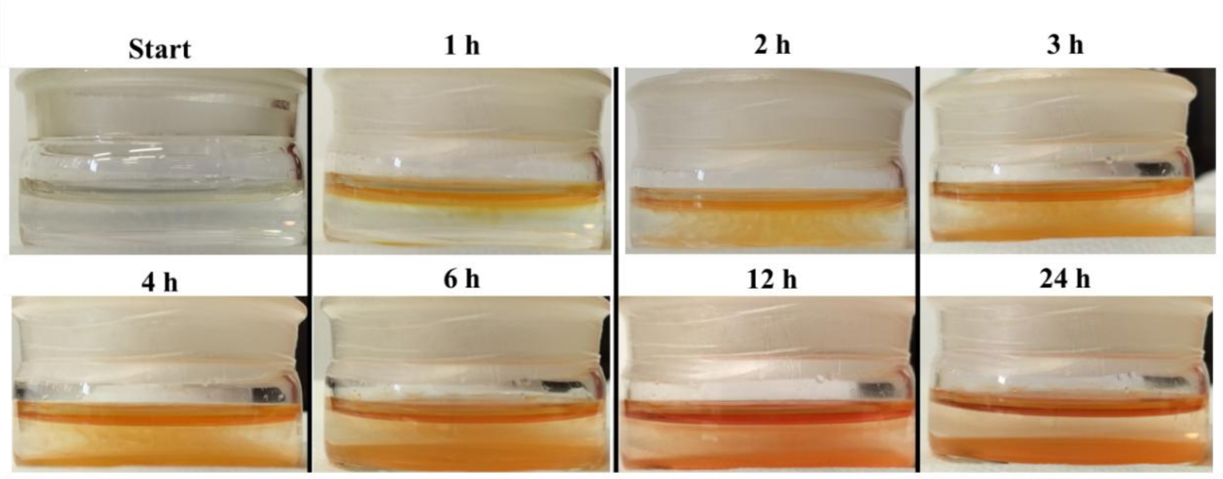

Figure S3. Photographic images showing the growth of TFP-PDA COF films. HAc was introduced in the organic phase along with the aldehyde monomer, TFP, while the PDA was present in the aqueous phase.

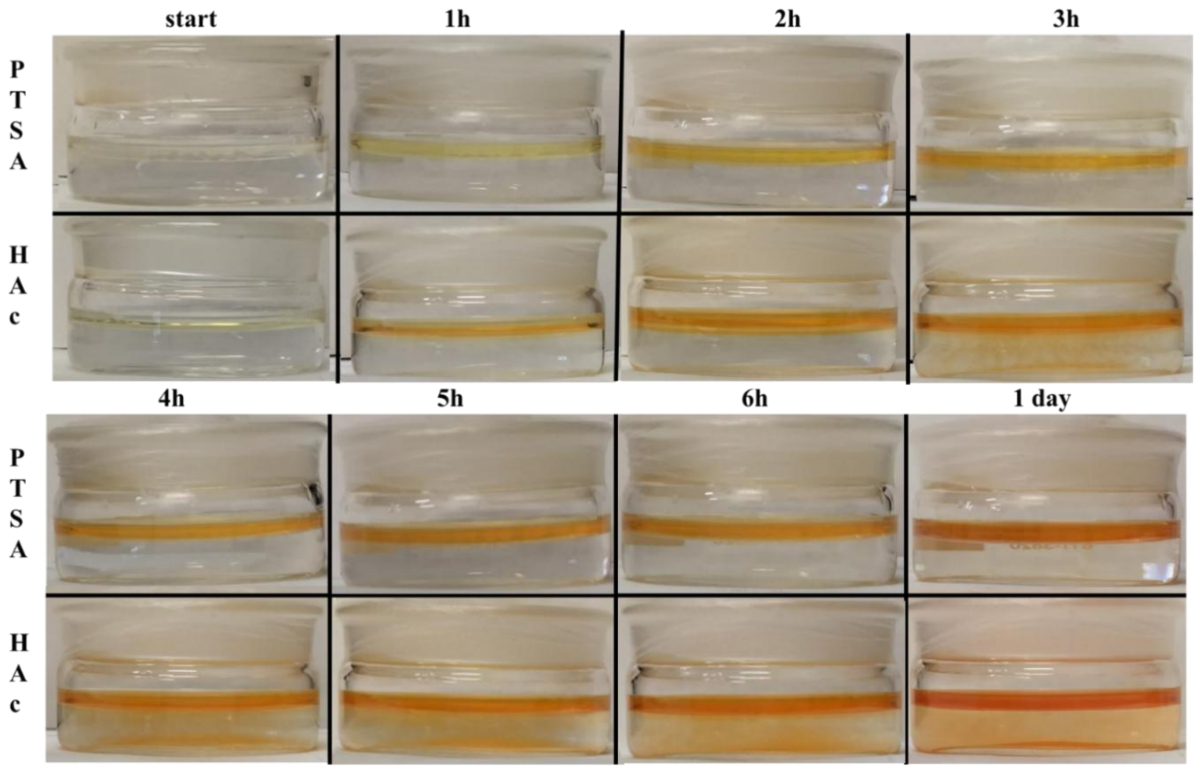

Figure S4. Photographic images showing the growth of TFP-PDA COF films. The catalyst, PTSA (top) or HAc (bottom), was introduced in the aqueous phase along with the amine monomer, PDA, while the TFP was present in the organic phase.

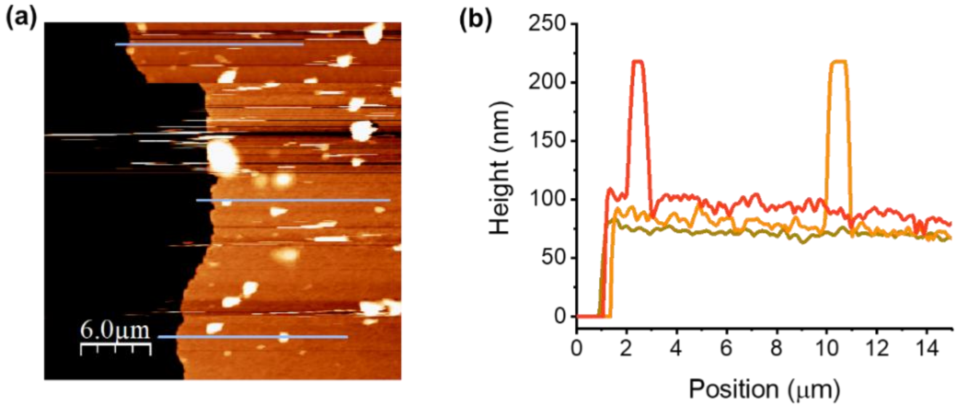

Figure S5. Tapping-mode AFM height image (a) and thickness profiles (b) of the TFP-PDA film deposited on a silicon substrate after 24 h reaction using HAc in the organic phase.

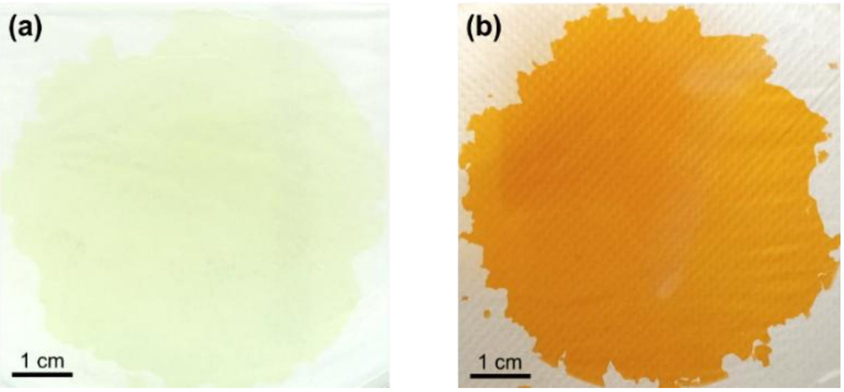

Figure S6. Photographic images of the as-synthesized TFB-PDA (a) and TFP-PDA (b) COF films obtained after 24 h reaction.

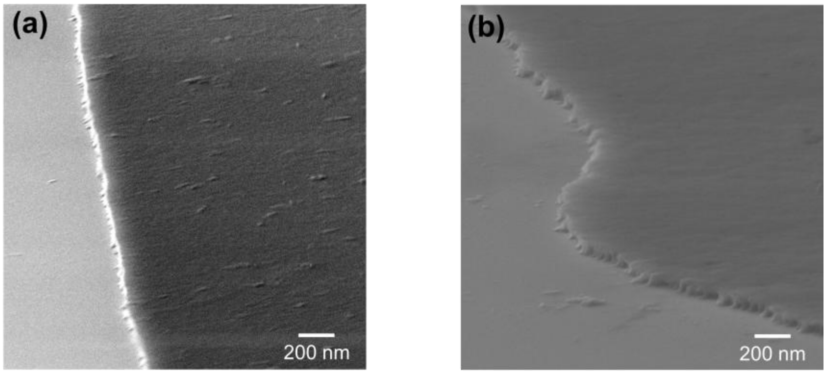

Figure S7. SEM images showing the surface morphology of the TFB-PDA (a) and TFP-PDA (b) COF films transferred on silicon substrates after 24 h reaction.

B. GISAXS

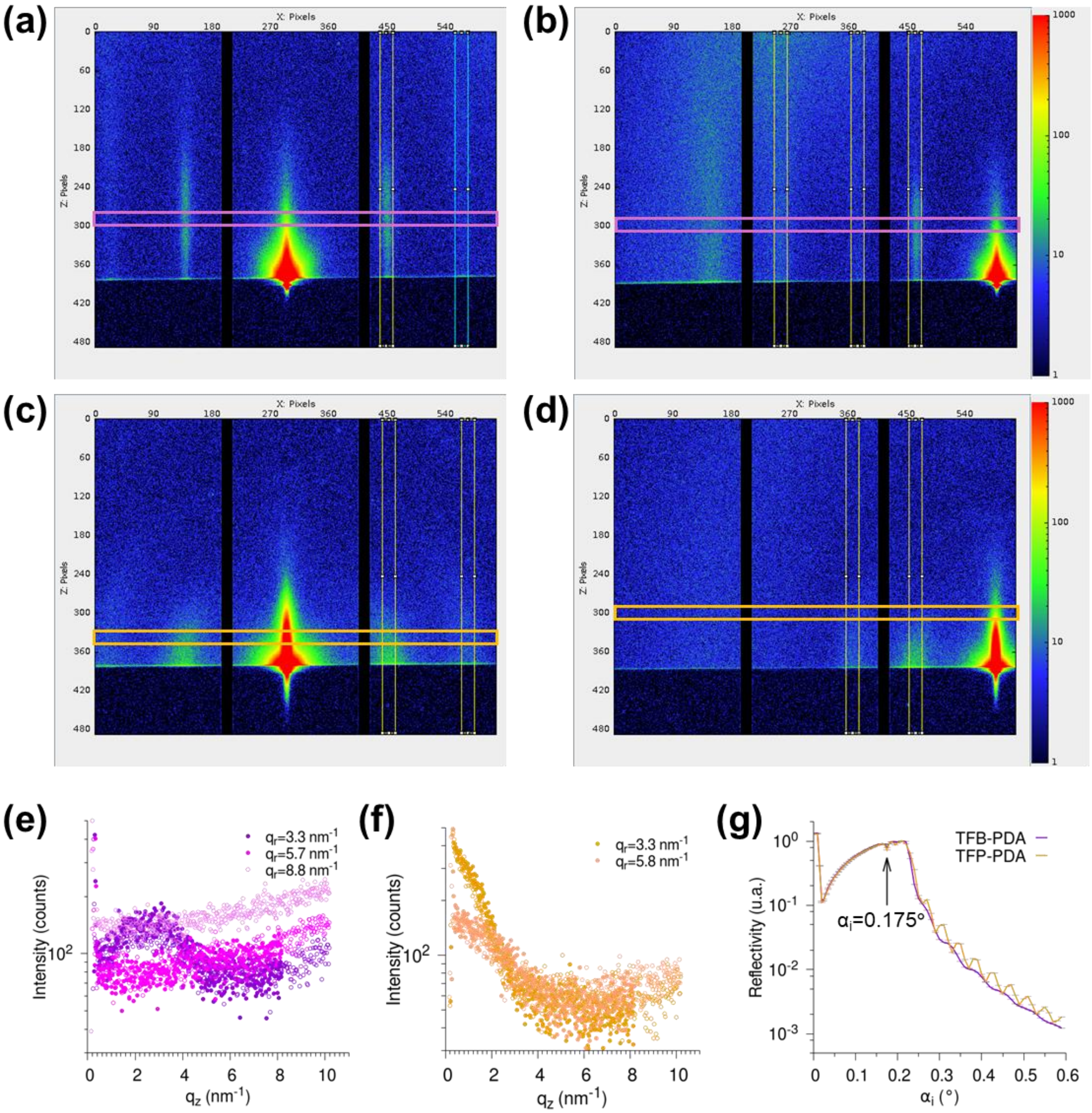

Figure S8. Raw 2D GISAXS images obtained with two detector positions for the synthesized TFB-PDA (a,b) and TFP-PDA (c,d) films, highlighting the regions included for integrating the horizontal (reported in the main text, as in-plane) and vertical, reported in panels (e) and (f), as out-of-plane, one-dimensional profiles. The X-ray reflectivity profiles are reported in (g).

The 2D scattering patterns were represented in the reciprocal space ($q_r = \sqrt{q_x^2 + q_y^2}, q_z$) considering the definitions $q_x = \frac{2\pi}{\lambda}(\cos(\alpha_f)\cos(2\theta) - \cos(\alpha_i))$, $q_y = \frac{2\pi}{\lambda}(\cos(\alpha_f)\sin(2\theta))$, and $q_z = \frac{2\pi}{\lambda}(\sin(\alpha_f)+\sin(\alpha_i))$, where α_i is the incidence angle on the surface plane, α_f is the out-of-plane exit angle from the sample horizon and 2θ is the in-plane exit angle. One-dimensional intensity vs. q_r (horizontal cuts) and q_z (vertical cuts) profiles are obtained using Python code from Eduardo Solano (NCD-SWEET beamline, ALBA Synchrotron Light Source), and peak positions and widths were evaluated by fitting with a Lorentzian peak function.

Table S1. Diffraction peak positions (q_{peak}) and half width at half maximum (HWHM) obtained from the GISAXS in-plane cuts, and comparison with peak positions reported in the literature (2θ, q_{calc}).

**TFB-PDA**						
***Literature*[a]**		***Experimental GISAXS***				
2θ- CuKα (°)	q_{calc} (nm^{-1})	q_{peak} (nm^{-1})	σ q_{peak} (nm^{-1})	HWHM (nm^{-1})	σ HWHM (nm^{-1})	d ($2\pi/q_{peak}$) (nm)
4.7	3.31	3.2854	0.0002	0.140	0.001	1.91
8.0	5.68	5.729	0.002	0.257	0.005	1.10
9.2	6.56	-				
12.2	8.68	8.817	0.002	0.294	0.005	0.71
16.1	11.38	11.451	0.002	0.675	0.004	0.55
24.7	17.43					
24.8	17.50					
**TFP-PDA**						
***Literature*[b]**		***Experimental GISAXS***				
2θ- CuKα (°)	q_{calc} (nm^{-1})	q_{peak} (nm^{-1})	σ q_{peak} (nm^{-1})	HWHM (nm^{-1})	σ HWHM (nm^{-1})	d ($2\pi/q_{peak}$) (nm)
4.7	3.34	3.303	0.001	0.542	0.005	1.90
8.3	5.90	5.810	0.001	0.84	0.02	1.08
11.1	7.88	-				
		11.39	0.01	1.51	0.05	0.55
27.0	19.02					

[a] Simulated powder x-ray diffraction as reported in ref. 1.

[b] Experimental powder x-ray diffraction as reported in ref. 2.

C. FT-IR Spectroscopy

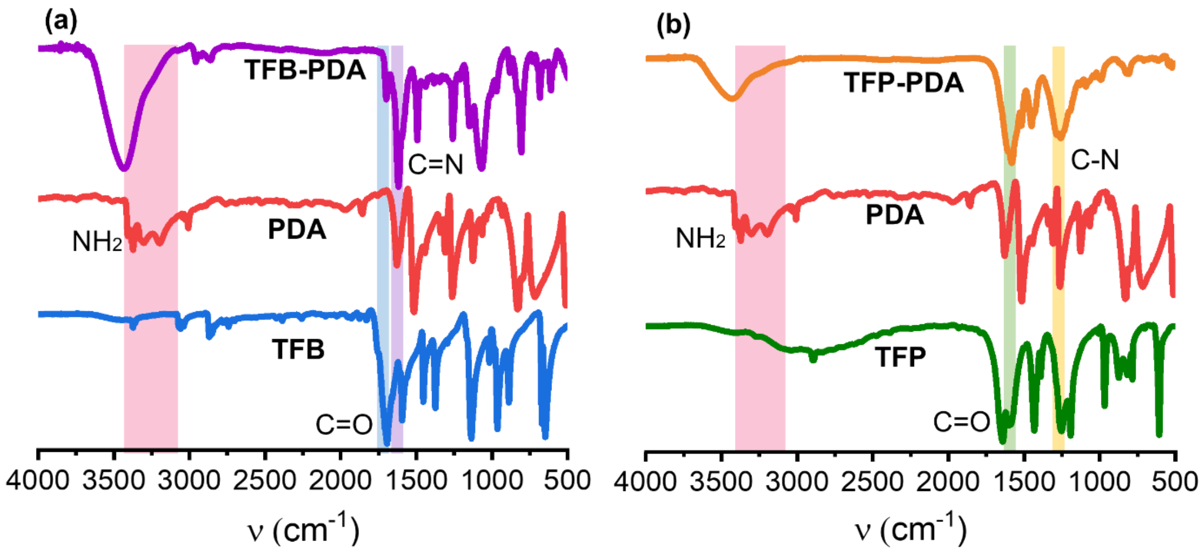

Figure S9. FT-IR spectra of TFB-PDA (a) and TFP-PDA (b) with the corresponding monomers.

D. UV-Vis Spectroscopy

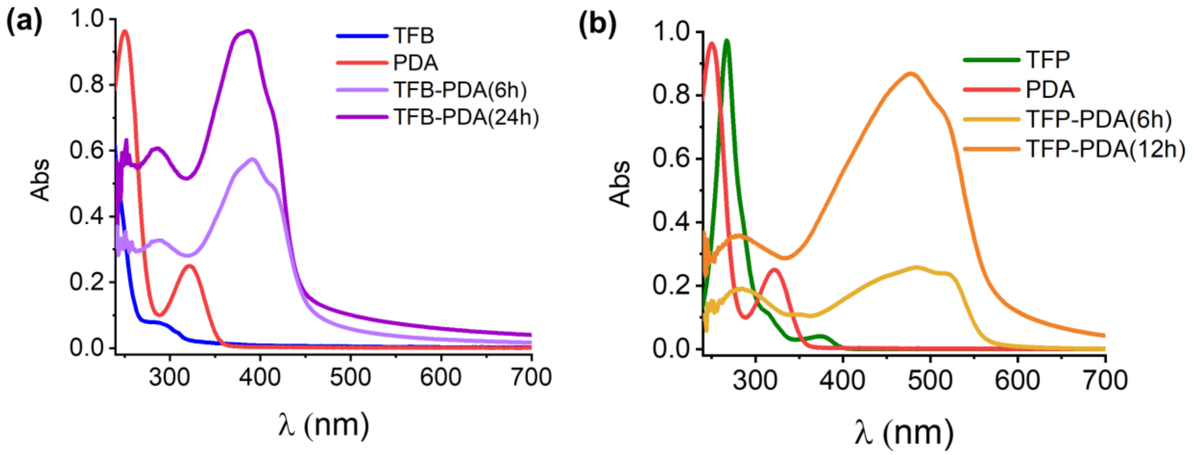

Figure S10. (a) UV-Vis absorption spectra of PDA (red) and TFB (blue) solutions and TFB-PDA film (purple) at different reaction times deposited onto a quartz substrate. (b) UV-Vis absorption spectra of PDA (red) and TFP (green) solutions and TFP-PDA film (orange) deposited onto a quartz substrate recorded at different reaction times.

E. X-ray Photoemission Spectroscopy

The elemental composition of the TFB-PDA COF obtained from the area of the different peaks (see the survey for a qualitative evaluation) is 81% C, 15% N and 4% O. The fraction of C and N is consistent with a stochiometric TFB-PDA COF while the presence of O with a binding energy (BE) of 532.1 eV compatible with hydroxyl oxygen (C-OH) can be partly due to surface contamination. The atomic percentages obtained from the XPS analysis of the TFP-PDA COF are 75% C, 11% N and 14% O, which are in good agreement with the atomic percentages obtained from the model of a non-defective polymer shown in Figure 4d.

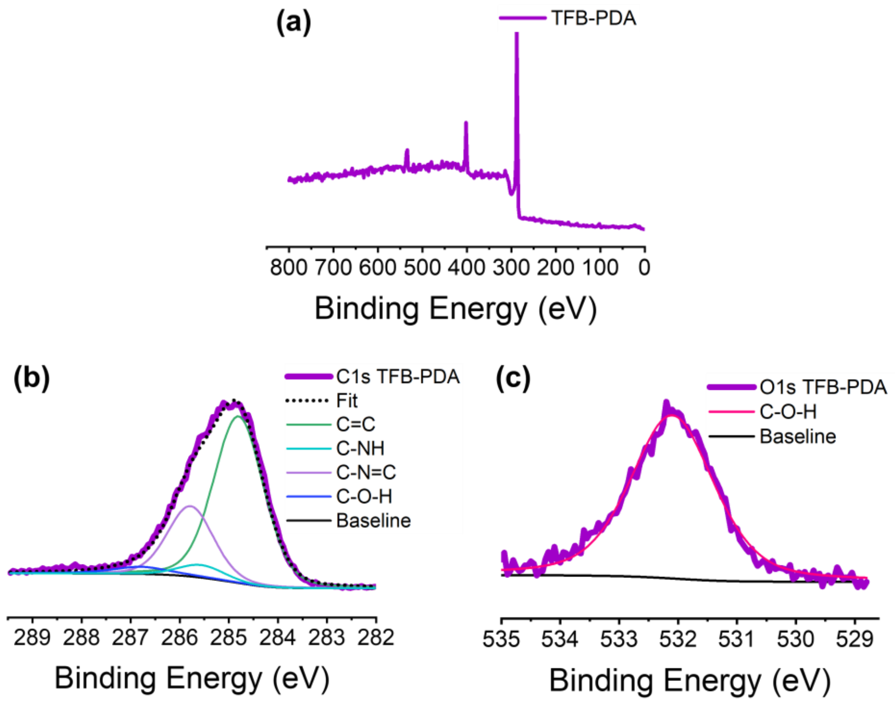

Figure S11. XPS survey spectrum of the TFB-PDA film (a). High-resolution XPS spectra of C 1s (b) and O 1s (c).

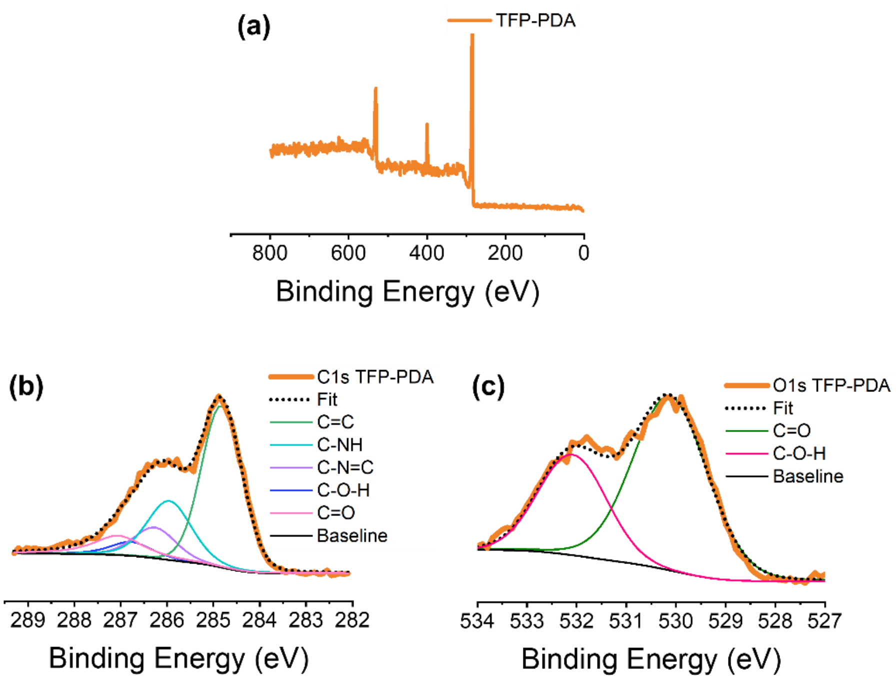

Figure S12. XPS survey spectrum of the TFP-PDA film (a). High-resolution XPS spectra of C 1s (b) and O 1s (c).

F. Nitrogen Adsorption Analysis

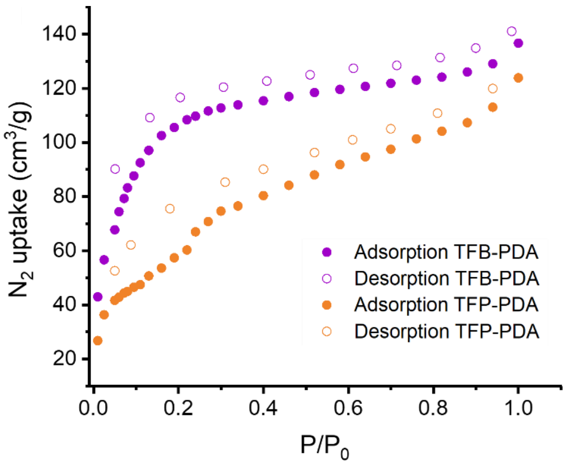

Figure S13. N_2 adsorption (closed circles) and desorption (open circles) isotherms of the TFB-PDA (purple) and TFP-PDA (orange).

G. Mechanical Characterization

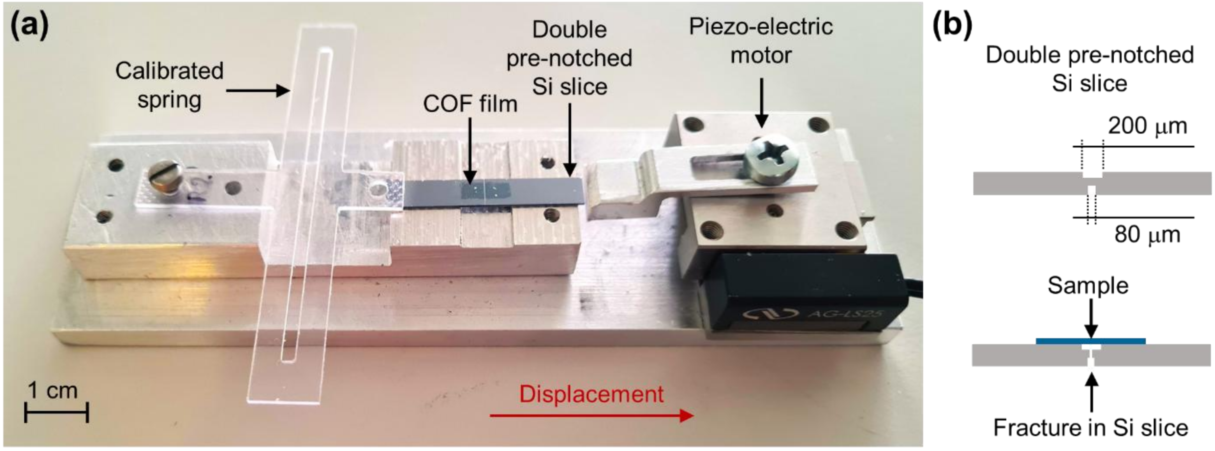

Figure S14. a) Image of the custom-made nanotensile platform employed for the mechanical characterization of COF films. b) Schematic illustration of a double pre-notched silicon slice, showing the dimensions of the top and bottom pre-notches. Fracture initiation occurs at the bottom pre-notch when pressure is applied using a sharp tip, causing the silicon slice to break into two pieces. The COF film region across the top pre-notch in the silicon slice is freestanding and corresponds to the area subjected to tensile testing.

H. Molecular Dynamics Simulations

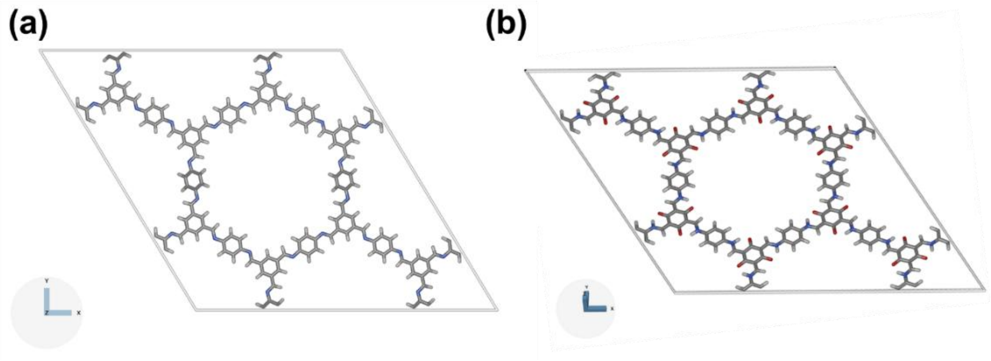

Figure S15. Starting configuration of 2x2 supercells of (a) TFB-PDA and (b) TFP-PDA, based on atomic coordinates in the CURATED database.[3,4]

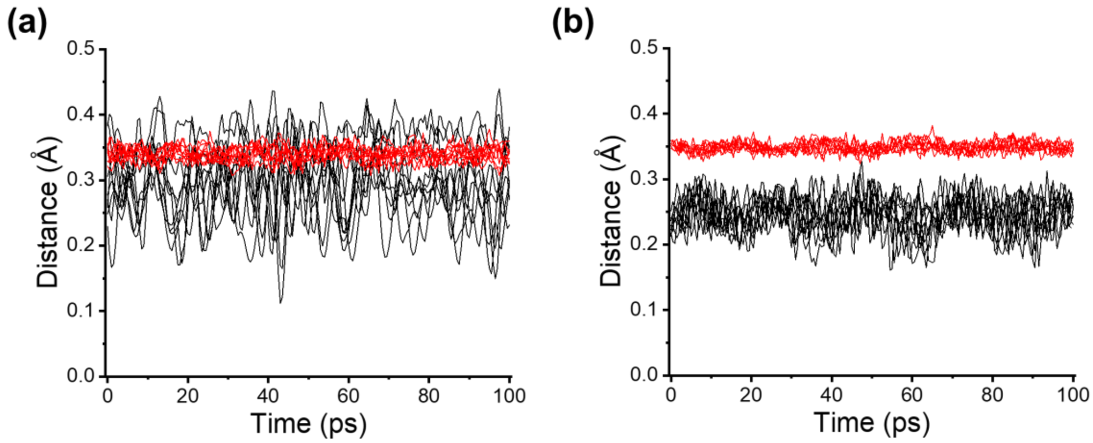

Figure S16. Longitudinal (red) and lateral (black) displacements between the centers of mass of adjacent 2x2 supercells, as a function of time in 100 ps NVT trajectories for 10 layers of (a) TFB-PDA and (b) TFP-PDA at 300K.

I. References

1. Ying, Y.; Peh, S. B.; Yang, H.; Yang, Z.; Zhao, D. Ultrathin Covalent Organic Framework Membranes via a Multi-Interfacial Engineering Strategy for Gas Separation. *Adv. Mater.* **2022**, *34*, 2104946.

2. Kandambeth, S.; Mallick, A.; Lukose, B.; Mane, M. V.; Heine, T.; Banerjee, R. Construction of Crystalline 2D Covalent Organic Frameworks with Remarkable Chemical (Acid/Base) Stability via a Combined Reversible and Irreversible Route. *J. Am. Chem. Soc.* **2012**, *134*, 19524–19527.

3. Ongari, D.; Yakutovich, A. V.; Talirz, L.; Smit, B. Building a Consistent and Reproducible Database for Adsorption Evaluation in Covalent-Organic Frameworks. *ACS Cent. Sci.* **2019**, *5*, 10, 1663–1675.

4. Ongari, D.; Talirz, L.; Smit, B. Too Many Materials and Too Many Applications: An Experimental Problem Waiting for a Computational Solution. *ACS Cent. Sci.* **2020**, *6*, 11, 1890–1900.
